# Unveiling Prophage Diversity and Host Interactions in Liberibacter: Genomic Insights for Phage Therapy Against Citrus Huanglongbing

**DOI:** 10.3390/biology14050576

**Published:** 2025-05-20

**Authors:** Hui Yin, Jiaxing Wan, Siyu Zhang, Zhuozhuo Wu, Wanshan Zhang, Yuxia Gao

**Affiliations:** 1 National Navel Orange Engineering Research Center, Gannan Normal University, Ganzhou 341000, China; yh@gnnu.edu.cn (H.Y.); wjx@gnnu.edu.cn (J.W.); zsiyu0022@163.com (S.Z.); three_kangaroo@163.com (Z.W.); zws@gnnu.edu.cn (W.Z.); 2 Jiangxi Provincial Key Laboratory of Pest and Disease Control of Featured Horticultural Plants (2024SSY04181), Ganzhou 341000, China

**Keywords:** Huanglongbing, prophage, virulence factors, diversity

## Abstract

HLB is a severe and devastating disease affecting citrus plants, for which no cure is currently available. The pathogen CLas is an unculturable, phloem-limited α-proteobacterium associated with HLB. Phages, known for their ability to directly lyse pathogenic bacteria, have been widely recognized as effective control agents in both medical and agricultural fields. In this study, we identified 191 prophages across 48 Liberibacter genomes using comparative genomics and phylogenetic methods. These prophages were classified into 17 distinct types, with 13 novel types, named NF1 to NF13. Diversity analysis revealed that the number of prophages per genome ranged from one to eight, with an average of four. Annotation showed that the genome sizes of NF1–NF13 ranged from 10,160 bp to 72,736 bp, with an average size of 27,616 bp, containing between 7 and 66 genes (average: 27 genes). Further functional enrichment of the sequences within 5 kb upstream and downstream of the integration site identified six main aspects, involving four core biological processes: genome structure modification, gene expression regulation, stress response activation, and metabolic pathway modulation. It is speculated that the insertion of the prophage significantly impacts the host. Six virulence factors, including *carB*, *clpC*, *flhA*, *algW*, *rffG*, and *IlpA*, were identified in the prophages. Interestingly, prophages containing *carB* or *clpC* are predominantly found in CLas, which may contribute to the global colonization of CLas. Notably, among the 35 identified Type 4 prophages, 19 were found to be active. Type 4 is the only active prophage type, making it a promising candidate for developing phage therapy against CLas. This study is significant for a deeper understanding of the diversity of Liberibacter and the interactions between prophages and their hosts.

## Simple Summary

Huanglongbing (HLB) is a destructive disease in citrus plants, putatively caused by the bacterium *Candidatus* Liberibacter asiaticus (CLas), which currently has no cure. In this study, we examined the genomes of 48 Liberibacter strains and identified 191 prophages, which are virus-like entities found within the bacterial genomes. These prophages were classified into 17 types, 13 of which were newly discovered. Our analysis showed that the number of prophages varied across genomes, and we identified six key biological processes impacted by these prophages, such as changes in gene expression and stress responses. Some prophages contained genes linked to virulence factors, potentially aiding in CLas’s global spread. Notably, 19 of the identified Type 4 prophages were active, suggesting their potential for developing phage therapy as a treatment for HLB. This research contributes valuable insights into the diversity of Liberibacter and how prophages interact with their host.

## 1. Introduction

Prophages, commonly known as phages, are viruses that specifically target bacteria and are the most abundant biological entities on Earth. With an estimated population of 10^31^ phages across various ecosystems, they play a crucial role in controlling bacterial populations and shaping microbial ecology [1]. Phages influence bacterial evolution through processes like transduction, which can aid in the dissemination of antibiotic resistance genes [2].

Phages exhibit a wide range of structural forms, including icosahedral, filamentous, and head–tail shapes, and their life cycles can be classified into lytic and lysogenic types. Lytic phages, also known as virulent phages, initiate the lytic cycle upon infecting a host bacterium, commandeering the host’s cellular machinery to produce new phages, which leads to the lysis and death of the bacterial cell [3]. In contrast, temperate phages (also named prophages) can enter the lysogenic cycle, integrating their genetic material into the host genome and remaining dormant until certain conditions trigger their switch to the lytic cycle [3]. Various external factors, such as streptomycin, high temperatures, and ultraviolet radiation, can induce significant proliferation, release, and lysis of prophages within certain host cells [4,5]. These inducible prophages are also known as active prophages.

Prophages, the quiescent forms of bacteriophages integrated into bacterial genomes, play a crucial role in microbial ecology and evolution. These genetic elements are widely distributed across various bacterial species, contributing significantly to bacterial diversity and adaptability [6,7]. Typically, prophages remain latent within the host genome, replicating passively alongside the bacterial chromosome. However, under certain environmental triggers—such as stress conditions or exposure to specific chemicals—prophages can excise from the bacterial genome, transition to the lytic cycle, and produce new phage particles [8,9]. For example, under heat shock conditions, certain prophages excise from the host genome of *Escherichia coli* and enter the lytic cycle, producing new phage particles. Similarly, exposure to UV radiation or chemical agents can trigger prophage induction in *Staphylococcus aureus*. Thus, prophages themselves serve as promising tools for the control of bacterial pathogens in various applications.

The interplay between prophages and their bacterial hosts is intricate and multifaceted. Prophages can confer advantages to their hosts through lysogenic conversion, wherein phage genes bestow new traits such as toxin production, antibiotic resistance, or enhanced metabolic capabilities. These attributes can bolster bacterial fitness and competitiveness across diverse environments [10,11]. Conversely, prophages can also impose fitness costs on the host, particularly through spontaneous induction, which may lead to cell lysis and a consequent decline in population [12,13].

Moreover, prophages can modulate host gene expression and genome stability. The integration of prophage DNA may disrupt host genes or regulatory regions, potentially altering host phenotypes. Additionally, prophages facilitate horizontal gene transfer, thereby promoting the dissemination of virulence factors (VFs) and antibiotic resistance genes (ARGs) within bacterial communities [14,15].

Bacterial diseases have long been a significant factor affecting crop health and yield in modern agricultural production. However, with the overuse of chemical pesticides, environmental concerns, and the emergence of pathogen resistance, there is an increasing need for environmentally friendly and sustainable plant protection methods. In this context, bacteriophages—natural bacterial viruses—have shown tremendous potential due to their unique biological characteristics. Phages can effectively control a wide range of plant bacterial diseases. For example, phage-based products have been applied in agricultural production to combat bacterial spot disease in tomatoes and peppers, fire blight in apples and pears, citrus canker, citrus bacterial spot disease, and soft rot in potatoes [16,17,18,19]. A 2024 study also reported a “seed coating technology” where newly isolated phages, Athelas and Alfirin, derived from soil and wastewater, were directly applied to seed surfaces. These phages effectively infect bacteria responsible for crop diseases, providing a biological control measure for early plant growth [20].

The genus Liberibacter encompassing several species of phloem-restricted, Gram-negative bacteria, has garnered significant attention due to its association with economically devastating plant diseases, particularly citrus HLB. CLas, *Candidatus* Liberibacter americanus (CLam), and *Candidatus* Liberibacter africanus (CLaf), which cause citrus HLB or citrus greening [21,22], and *Candidatus* Liberibacter solanacearum (CLso), responsible for potato zebra chip disease, pose significant threats to global citrus and potato production, respectively [23]. *Liberibacter crescens* (Lcr) is the only single isolate of Liberibacter that has been cultured [24].

The initial discovery of Liberibacter was intertwined with efforts to understand HLB, a disease that severely impairs citrus production by causing yellowing of leaves, stunted growth, and fruit drop [25]. HLB was first reported in China in the early 20th century and later identified in other citrus-growing regions. The causal agents, primarily CLas, were characterized by their persistent colonization of the phloem tissue, making them difficult to study and eradicate [21].

The discovery of prophages in Liberibacter species, particularly CLas, has provided new insights into the genetic diversity and potential virulence mechanisms of these bacteria. For example, prophages SC1 (NC_019549.1, Type 1), SC2 (NC_019550.1, Type 2), and P-JXGC-3 (KY661963.1, Type 3) in CLas have been shown to contribute to genetic variability and may influence bacterial fitness and adaptability in different environmental conditions [26]. Moreover, genomic analyses have identified multiple prophage regions within the genomes of CLas. These prophages exhibit a range of genetic configurations and functional potentials, suggesting that they play diverse roles in bacterial ecology and evolution [27,28].

Prophage diversity in Liberibacter species is significant, and may reflect the complex interactions between these bacteria and their phage predators. Recent advances in high-throughput sequencing and bioinformatics have facilitated the comprehensive characterization of prophages in Liberibacter species. SC1, SC2, P-JXGC-3, Novel microviridae phage (CLasMV1), and Type 4 prophages have been identified in the CLas genome [26,29,30,31]. But it seems that the diversity of prophages in liberibacter is far from thoroughly analyzed. Based on this, this study utilizes bioinformatics-related methods to identify novel prophage sequences. We identified a total of 13 novel prophages from 48 Liberibacter genomes which significantly expanded their diversity. Additionally, we found that the majority of Type 4 prophages are active, suggesting their potential as candidate phages for the treatment of CLas. This is of significant importance for novel approaches for controlling HLB and developing effective disease management strategies.

## 2. Materials and Methods

### 2.1. Data Used in This Study

A total of 48 genomes from Liberibacter species were selected for this study. This selection includes 34 CLas, 2 CLam, 2 CLaf, 2 Lcr, and 8 CLso genomes (Table S1). The genome data were downloaded from the NCBI database (https://www.ncbi.nlm.nih.gov/datasets/genome/?taxon=34019) (accessed on 9 September 2024).

### 2.2. Indentification of Prophage in Liberibacter

Prophage Hunter [7] software with the “Skip similarity matching” option was used to predict prophages in the Liberibacter genome. The results from the “03.Closest_phages.Candidate” file indicated BLASTX results, retaining candidate prophage predictions with an E-value < 10^−3^. The candidate prophage sequences and their corresponding closest phages were obtained. The protein sets of the closest phages were downloaded from the NCBI database and a database was constructed. BLASTX searches were performed on the candidate prophage sequences to identify hallmark genes.

Protein sequences of hallmark genes from reference species (bacteria, archaea, fungi, virus, plant, animal) were downloaded from the NCBI protein database. Multiple sequence alignments of the hallmark genes of candidate prophages and their homologous sequences were performed using the L-INS-i algorithm in the MAFFT V. 7.463 [32] software. IQ-TREE v.2 [33] software was used to automatically detect and select the optimal substitution model with the -m MFP parameter, followed by constructing a maximum likelihood phylogenetic tree using the -bb 1000 parameter. Candidate prophages that formed a monophyletic group with known viruses in the phylogenetic tree were identified as prophages of Liberibacter.

### 2.3. Genome Annotation of Prophages in Liberibacter

To annotate the protein functions of Liberibacter prophages, we used the Prokka [34] software with default parameters. To identify potential virulence factors (VFs), the fasta format sequences of the prophage genomes were submitted to the “Search” tool in the Virulence Factors Database (VFDB) (http://www.mgc.ac.cn/VFs/) (accessed on 15 April 2025) [35] under the core datasets SetA and SetB for blastn analysis. To elucidate the impact of phage integration on host genomic architecture, we extracted 5 kb genomic regions flanking both the upstream and downstream of the insertion site. Functional annotation of these sequences was systematically performed using eggNOG-mapper v2.1.12 (parameters: -m diamond -d bact) [36] for evolutionary genealogy analysis, followed by Gene Ontology (GO) term assignment through TBtools v2.098 (E-value < 1 × 10^−5^, Fisher’s exact test with FDR correction) [37] to characterize molecular functions and biological processes.

### 2.4. Phylogenetic Analysis

Homologous gene clusters in 48 Liberibacter genomes were identified using the PEPPAN software [38]. The structural annotation files (GFF) of the 48 genomes were used as input files. The pan-genome was constructed by executing the command PEPPAN -t 8 -p result/peppan./gff/*.gff. To analyze the pan-genome, the following command was used: PEPPAN_parser -g result_peppan/peppan_PEPPAN.gff -s result_peppan/PEPPAN_out -t -c. Based on the presence–absence profiles of all pan genes, a phylogenetic tree of Liberibacter species was generated.

### 2.5. PCR Amplification and Prophage Cloning

*Lcr* str. BT-1 was preserved in our laboratory. Primers were designed for the hallmark gene sequences (Figure 3) of four identified prophages: NF2, forward: ATGGATAT AGCTAAATATTCAGAGCTTGTACGTAG, reverse: CTAAGCTGTTGAAAATT CCTCCTTAGAATTTTCATATG; NF6, forward: ATGACTGAAAAAAATCATAGTGTAACATCTTTATACCG, reverse: TCAGCGATCATATTGATGATGATGATAAGC; NF7, forward: ATGAAAGGTATAATTCTTGCTGGAGGAAATGG, reverse: TAAAAAATGCTTTTTAAATATTGTCCATAAAGATTATTTCCATAG; NF8, forward: ATGATTAAGAAAAAGCTTCCTAGAGTTATATTTCATACACG, reverse: ATGATTAAAGAAAAAGCTTCCTAGAGTTATATTTCATACACG. DNA was extracted from the Lcr str. BT-1 culture using the Rapid Bacterial Genomic DNA Isolation Kit (Sangon Biotech) (Shanghai, China). A total of 1 uL DNA, 1 uL primer, and the high-fidelity enzyme PrimeSTAR™ Mix (TaKaRa) (Beijing, China) were mixed and then a PCR amplification was performed on the PCR instrument (C1000 Touch Thermal Cycler, Bio-Rad, Hercules, CA, USA) with 1 min of denaturation at 95 °C, 30 s of annealing at 75 °C, and 1 min of extension at 72 °C. After 40 cycles of the above process, the PCR product was obtained and agarose gel electrophoresis for detection was performed. DNA fragments of the appropriate sizes were excised and ligated into a T-vector (pEASY-Blunt Cloning Kit, Transgen) (Beijing, China) before being transformed into DH5α competent cells. After 18 h at 37 °C, the plaque was added to a liquid culture medium for reproduction. Colony PCR was conducted for positive detection, and positive colonies were sent to a sequencing company for Sanger sequencing.

## 3. Results

### 3.1. The Diversity of Prophage in Liberibacter

**Figure 1:**
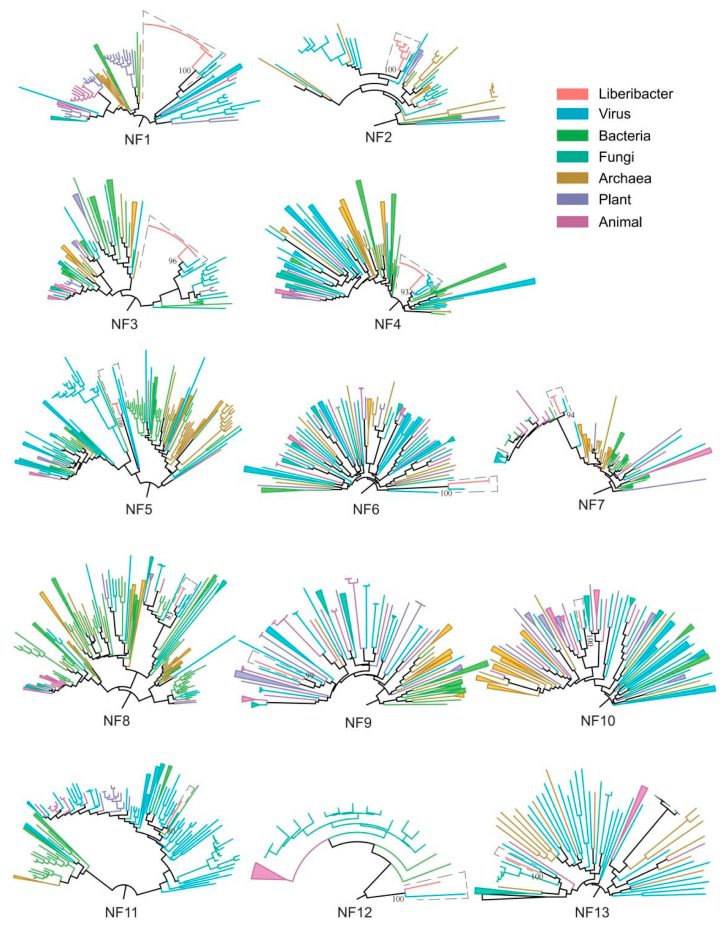
The phylogenetic relationships of hallmark proteins. The prophage sequences are enclosed in dashed boxes on each phylogenetic tree. In each tree, the phage sequences form monophyletic clades and are closely related to viruses. Bootstrap values are depicted near the branches with the closest evolutionary relationships.

This study integrates comparative genomics and phylogenetic approaches to identify prophages within 48 Liberibacter genomes. Using the Prophage Hunter software, 61 putative prophages (59 novel and 4 reported) were identified across 48 Liberibacter genomes Comparative genomics helped in identifying hallmark genes. Phylogenetic analysis based on hallmark genes revealed that 13 newly found putative prophages and viruses form a monophyletic group (Figure 1). These 13 putative prophages were classified as newly found (NF) Liberibacter prophages and were named NF1, NF2, NF3, NF4, NF5, NF6, NF7, NF8, NF9, NF10, NF11, NF12, and NF13. In total, we identified 13 novel Liberibacter prophages along with 4 previously reported prophages (Type 1, Type 2, Type 3 and Type 4), expanding the number of Liberibacter prophages from 4 to 17.

**Figure 2:**
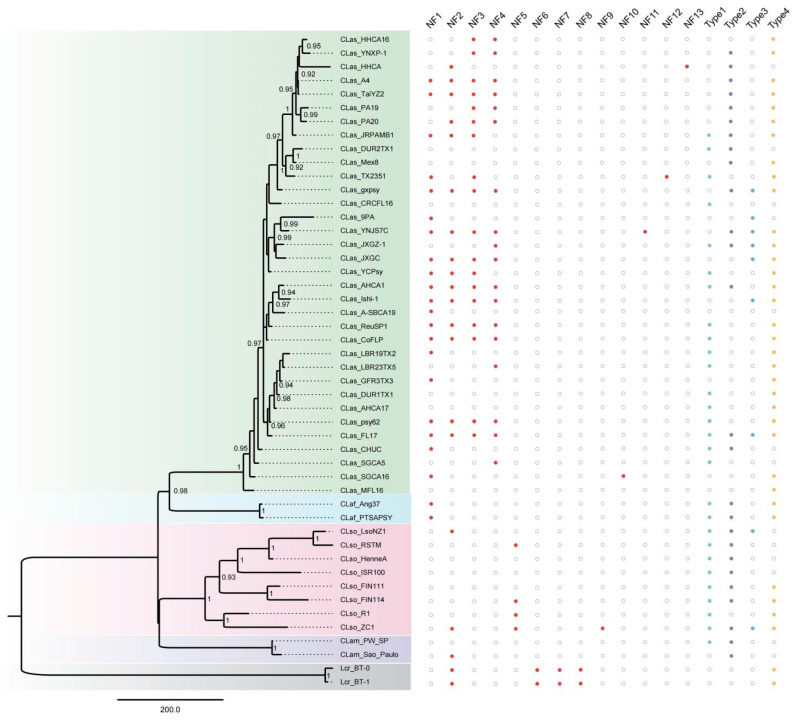
The diversity of prophages identified in 48 Liberibacter genomes. The phylogenetic relationships of 48 Liberibacter genomes are displayed on the left, with the tree root determined using the Midpoint method [39]. Bootstrap values are indicated next to the corresponding nodes. Solid circles represent presence, while hollow circles represent absence. Different colors denote different phage types: red, green, purple, blue, and yellow correspond to novel phages, Type 1, Type 2, Type 3, and Type 4, respectively.The four colors on the phylogenetic tree represent the genomes of the four species of Liberibacter.

A total of 191 prophages were identified, and their distribution across the Liberibacter genomes was uneven (Figure 2, Tables S2 and S3). Novel prophages were identified based on the closest virus, revealing that, except for NF9, classified as Megaviricetes, and NF11, classified as unknown, the remaining 11 prophages were all classified as Caudoviricetes, consistent with the four previously reported phages (Table S3). NF1–NF4, along with Type 1–Type 4, were widely distributed across the genomes, whereas NF5 to NF13 were detected in only a few or single genomes (Figure 2). The number of prophages per genome ranged from one to eight, with an average of four. Both CLas str. FL17 and CLas str. YNJS7C contained eight prophages each, whose lengths accounted for 13.5 and 16.4% of the total genome lengths, respectively.

The CLas genomes, with the highest representation in this study (34 out of 50), also exhibited the greatest diversity. Among the prophages NF1 to NF4, all four were present in 10 genomes (Table S2). Interestingly, these 10 genomes were fully assembled into chromosomes, while genomes not assembled into chromosomes only partially contained these prophages (Table S1). This finding highlights the significant influence of genome assembly on phage identification, suggesting that these four prophages may be distributed across all CLas strains.

**Figure 3:**
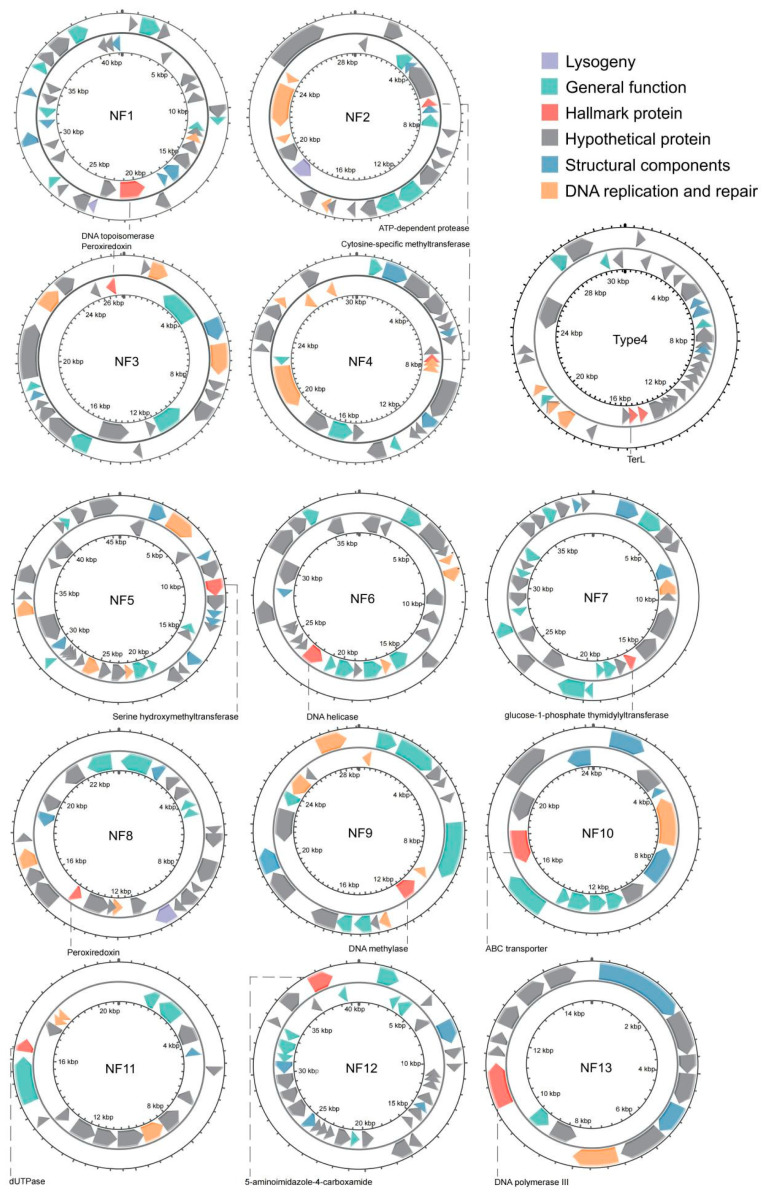
The annotations of 13 representative novel phage genomes. Blocks of different colors represent various types of proteins, with each phage featuring distinct hallmark proteins. Dashed lines connect to the specific names of the hallmark proteins.

Four newly found prophages (NF2, NF6, NF7, and NF8) were identified within the Lcr str. BT-1 genome. We designed primers to amplify four hallmark gene sequences (Figure 3), with lengths of 861 bp (NF2), 531 bp (NF6), 2619 bp (NF7), and 1473 bp (NF8) (Figure S1A). DNA fragments of the correct sizes were extracted from the agarose gel, cloned into a T-vector, and transformed into the *Escherichia coli* strain DH5α. Positive colonies were validated by PCR, and those confirmed as positive were sent for sequencing (Figure S1B). Sequencing results confirmed that the amplified sequences matched those detected in the genome, demonstrating the accuracy and high reliability of our identification.

### 3.2. Genome Structure of Novel Prophage

The length of the NF1 to NF13 phage genomes ranged from 10,160 to 72,736 bp, with an average of 27,616 bp. The number of genes varied from 7 to 66, with a mean of 27. The GC content was approximately 36% (Table S3). Notable length discrepancies were observed among phage genomes of different species; for example, 22 NF1 genome sequences were sourced from 20 CLas and 2 CLaf genomes. The lengths of the two NF1 genomes from CLaf were approximately 65,510 bp, while the NF1 lengths of 20 CLas genomes averaged only 24,040 bp. Furthermore, the genome lengths of the 20 NF1 displayed a more complex distribution, consisting of 13 at 18,847 bp, two at 27,767 bp, one at 27,761 bp, one at 33,561 bp, one at 37,788 bp, one at 38,801 bp, and one at 42,322 bp (Table S3). It can be inferred that phages of identical length originated from a singular insertion event. Based on the closest phage, 11 navel prophages were identified as Caudoviricetes viruses, consistent with four reported phages. NF9 was classified as a Megaviricetes viruses, while NF11 remained unclassified.

To conduct a more in-depth analysis, we selected representative genomes from 13 newly identified phages (Table 1). Protein-coding sequences (CDS) were annotated using Prokka software. The proteins were classified into six categories: lysogeny, general function, hallmark proteins, structural components, DNA replication and repair, and hypothetical proteins (Figure 3). Proteins involved in the process of prophages infecting bacteria and integrating their genomes into the host bacterial chromosome were uniformly classified into the lysogeny category. This category included lysin B and holin. Lysogeny-related proteins were identified only in the genomes of NF1, NF2, and NF8. The general function category consisted of enzymes involved in viral activities, such as metallophosphoesterase, glutamine amidotransferase, glycosyl transferase, UTP-glucose-1-phosphate uridylyltransferase, and esterase. Structural components include proteins that maintain the viral particle, such as structural proteins, tail length tape-measure proteins, major head proteins, minor capsid proteins, tail fiber hinges, and tailspike proteins. Proteins involved in DNA replication and repair were classified into a separate category. Key examples included DNA polymerase, DNA helicase, DNA topoisomerase, DNA recombinase, and DNA-binding proteins. Proteins used to identify prophages and build phylogenetic relationships were categorized as hallmark proteins. Each of the 13 prophages had distinct hallmark proteins. For NF1 to NF13, these were DNA topoisomerase, ATP-dependent protease, peroxiredoxin, cytosine-specific methyltransferase, serine hydroxymethyltransferase, DNA helicase, glucose-1-phosphate thymidylyltransferase, peroxiredoxin, DNA methylase, ABC transporter, dUTPase, 5-aminoimidazole-4-carboxamide, and DNA polymerase III. Lastly, hypothetical proteins refer to those with unknown functions.

### 3.3. Prophage Impact on Host Organisms

**Figure 4:**
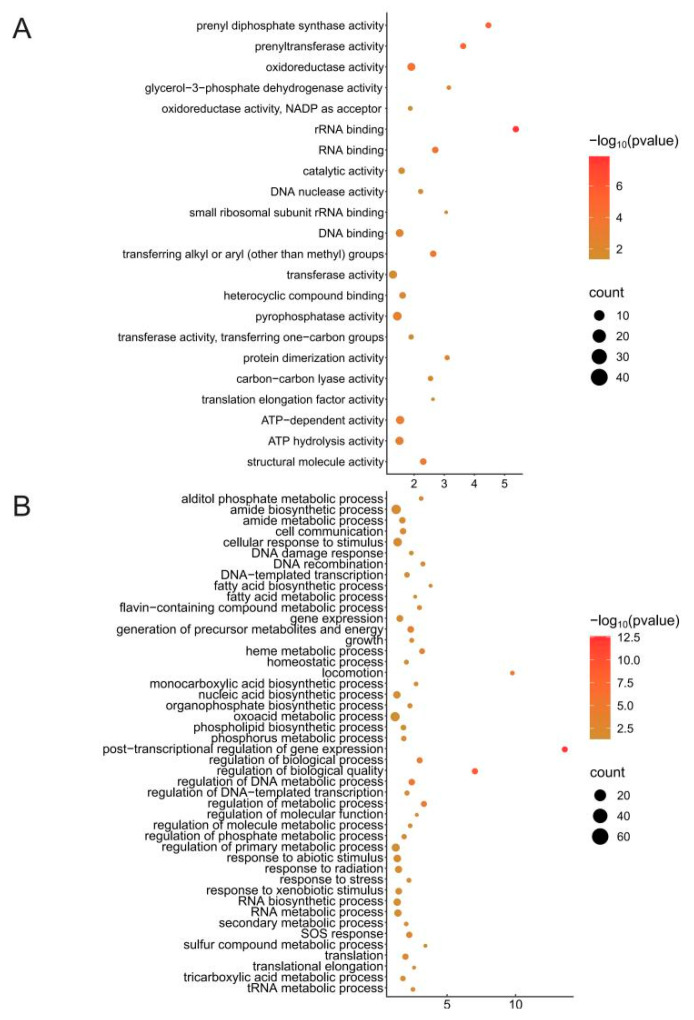
Functional enrichment analysis of 5 kb sequences upstream and downstream of the prophage integration site. (**A**): Molecular function enrichment results. (**B**): Biological process enrichment results. The X-axis represents Enrichment Score, and the Y-axis represents the molecular functions (**A**) and biological processes (**B**) enriched with *p* values less than 0.01. The size of the balloon in the figure represents the number of each category.

When a protophage is inserted into the host genome through site-specific recombination (e.g., integrase-mediated attB/attP site integration), it may directly destroy or alter host genes and may significantly affect the production of accessory genes at integration sites. In order to explore the possible influence of the protophage on Liberibacter, we extracted the 5 kb sequences upstream and downstream of the integration site for functional enrichment analysis, and there were 22 molecular functions, mainly including six aspects (Figure 4A). They were energy metabolism and ATP use of ATP hydrolysis activity; ATP-dependent and oxidoreductase activity; protein synthesis and translation regulation of translation elongation factor activity, small ribosomal subunit rRNA binding, and RNA binding; DNA metabolism and genome stability of DNA binding, DNA nucleotide activity, and catalytic activity; metabolic pathways and signal transduction of transferase activity, pyrophosphatase activity, and heterocyclic compound binding; structural molecule activity and protein dimerization activity; prenyltransferase and prenyl diphosphate synthase activity; and lipid and isoprenoid metabolism (Figure 4A). There are 29 biological processes that may be affected, mainly four core aspects (Figure 4B). They are basic metabolism and energy supply, such as the tricarboxylic acid metabolic process, fatty acid metabolic process, and translational elongation; environmental stress and damage repair, such as response to xenobiotic stimulus, response to radiation, SOS response, and DNA recombination; dynamic regulation of gene expression, such as regulation of DNA-templated transcription, post-transcriptional regulation, and regulation of the phosphate metabolic process; and the maintenance and adaptation of cellular functions, such as the phospholipid biosynthesis process, homeostatic process, and growth (Figure 4B). In conclusion, it is speculated that the integration of protophages may affect the energy metabolism, protein synthesis, DNA stability, metabolic pathways, and cell structure of host bacteria, and further promote the genome structure modification, gene expression regulation, stress response activation, and metabolic pathway adjustment of host bacteria.

Frequent horizontal gene transfer (HGT) occurs between protophage and host. The host may enhance its virulence by using virulence factors derived from the protophage. In this study, virulence factors in all identified prophages of Liberiacter were annotated. NF1, 3, 4, 12 and Types 1–4 are carbamoyl phosphate synthase large subunits (*carB*) (Table 2). Pyrimidine is essential for nucleic acid synthesis. This gene may help host bacteria to proliferate in nutrient-poor host environments and indirectly support phage replication. NF2, 10, and 13 were identified as endopeptide Clp ATP-binding chain C (*clpC*) (Table 2). The Clp system helps bacteria cope with oxidation, heat, or antibiotic stress. Phages may use this gene to enhance the survival rate of host bacteria in harsh environments and promote their own transmission. Interestingly, *carB* and *clpC* are mainly found in prophages distributed in CLas. Compared with CLam and CLaf, CLas has the most global distribution and causes the greatest harm. Previous studies have reported that *carB* is essential for canker development by *Xanthomonas citri* subsp. *citri*, and *ClpC* has been shown to be involved in the regulation of virulence in *clostridioides difficile* [40,41]. It is speculated that *carB* and *clpC* may be some of the factors that enable CLas to survive in many regions around the world.

Flagellar biosynthesis protein (*flhA*) was identified in the NF5 genome (Table 2). Flagella is the key to bacterial motility and host tissue colonization. Prophages may enhance the invasion ability of host bacteria through this gene. The AlgW protein in the NF6 genome regulates alginate synthesis and biofilm formation. Biofilms enhance bacterial resistance to antibiotics and host immunity. Prophages may assist host bacteria to form persistent infection in the host by transmitting this gene. dTDP-glucose 46-dehydratase (*rffG*) and immunogenic lipoprotein A (*IlpA*) were found in the NF7 and NF8 genomes, respectively. *rffG* is involved in lipooligosaccharide (LOS) synthesis and immune regulation. LOS is an important component of the outer membrane of Gram-negative bacteria and may help bacteria evade immune recognition. Prophages carrying this gene may enhance the immune evasion ability of host bacteria and promote infection. IlpA is a class of immune proteins involved in adhesion. Adhesion is the first step in infection, and prophages may help host bacteria colonize host cells more efficiently by delivering *IlpA*. Virulence genes may be integrated into host genomes through prophage horizontal gene transfer, which directly or indirectly enhances virulence (e.g., adhesion, immune escape, metabolic adaptation).

### 3.4. Type 4 Prophage as a Candidate Phage Against CLas

The Type 4 prophage was first identified in 12 CLas genomes in 2019 (29). In this study, we detected prophage sequences in the majority of the genomes (35/48), significantly increasing its diversity. In addition to CLam, this prophage was present in all CLaf (2/2) and Lcr (2/2) genomes, as well as in the vast majority of CLas (27/34) and CLso (4/8) genomes (Table 3). Moreover, only two CLam genomes were used in this study, so the absence of the Type 4 prophage in CLam cannot be conclusively determined. Overall, the Type 4 prophage is widely distributed across Liberibacter species.

**Figure 5:**
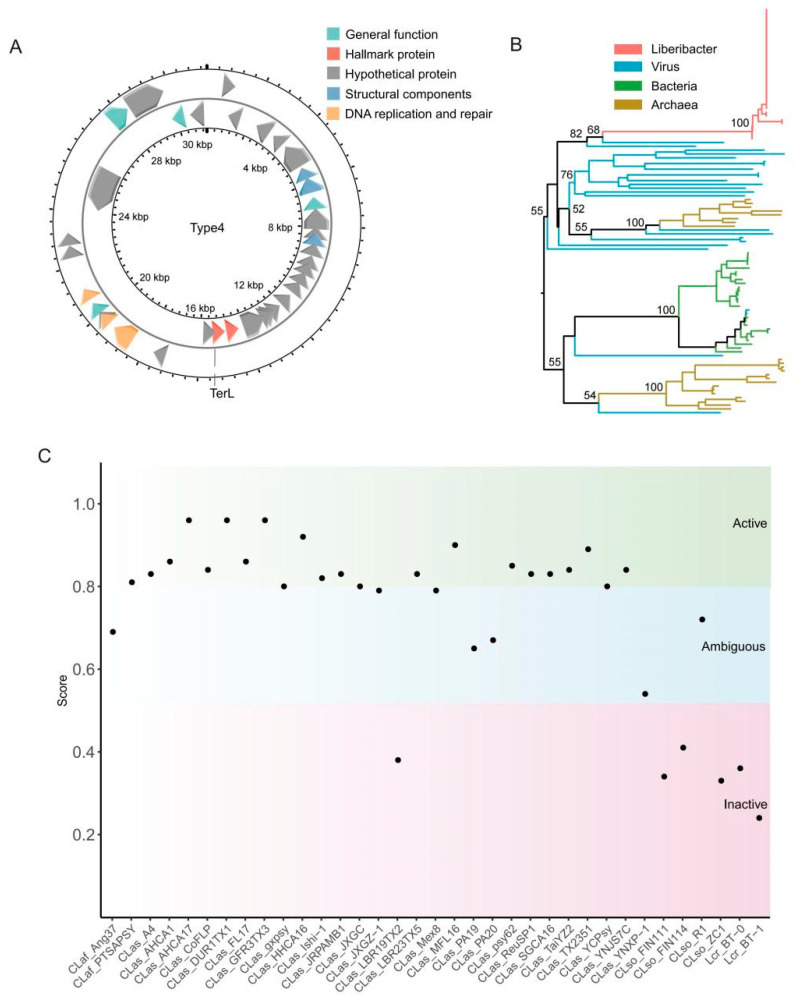
Information on the Type 4 phages identified in this study. (**A**): Genome annotation map of Type 4 phages. The genome of CLas str. A4 was annotated as a representative genome of Type 4 phages. The hallmark protein is TerL. (**B**): Phylogenetic tree based on the TerL protein, where red branches represent TerL proteins from Liberibacter, forming a monophyletic group with viruses. (**C**): Distribution chart of phage activity scores. Phages with scores above 0.8 are classified as active, those between 0.5 and 0.8 as ambiguous, and those below 0.5 as inactive.

The annotated Type 4 prophages range in full length from 11,096 to 54,972 bp, with an average length of 30,931 bp (Table 3). The genome structure was annotated, using the CLas A4 strain as a model, revealing four main categories: DNA replication and repair, structural components, general functions, and hypothetical proteins. The hallmark protein identified was TerL (Figure 5A). A phylogenetic tree based on the TerL protein demonstrated that Type 4 prophage sequences cluster together, forming a monophyletic group with viruses, confirming that these Liberibacter sequences are viral in origin (Figure 5B).

The Prophage Hunter software [7] scores the activity of each identified prophage sequence, with scores below 0.5 classified as inactive, between 0.5 and 0.8 as ambiguous, and between 0.8 and 1.0 as active. In this study, 19 active prophages were identified, all of which were of Type 4. Among the 35 Type 4 prophages, 19 were active, 10 were ambiguous, and 6 were inactive (Figure 5C). Of the 34 CLas genomes, 27 contained Type 4 prophages, including common ones like CLas_psy62 and CLas_A4. Moreover, except for the LBR19TX2 strain, which was inactive, the remaining 26 prophages were either ambiguous or active. Five genomes had scores above 0.9, with three (CLas_AHCA17, CLas_DUR1TX1, CLas_GFR3TX3) reaching the highest score of 0.96 (Table 3). These results suggest that Type 4 prophages may be promising candidates for combating CLas and warrant further investigation.

## 4. Discussion

This study utilized comparative genomics and phylogenetic methods to mine prophages from 48 Liberibacter genomes. Our analysis identified 13 new prophages, increasing the total number of known prophages associated with Liberibacter from 4 to 17. Previous research had only identified a limited number of phages in a small number of genomes. For instance, the initial report on Type 4 identified a few prophage-like elements in 15 CLas genomes [29]. In contrast, our study conducted a comprehensive prophage identification across 48 genomes (34 CLas, 2 CLam, 2 CLaf, 2 Lcr, and 8 CLso). In summary, this research provides a more detailed exploration of prophages in a broader range of genomes, identifying a greater diversity of phages, which is crucial for understanding the prophage diversity associated with Liberibacter.

This study used a more rigorous approach by combining comparative genomics with phylogenetic methods for virus-like sequence mining. Compared to Virsorter and PHASTER, Prophage Hunter is more sensitive in identifying viral sequences within genomes [7]. We identified 62 candidate prophage sequences. Since sequence similarity does not always reflect evolutionary relationships, we constructed a phylogenetic tree using hallmark genes of phages. Only candidate prophage sequences with the closest evolutionary relationships to viruses were considered prophages, eliminating false positives. Among the four bacterial species, Lcr str. BT-1 is cultivable. PCR amplification of the prophage sequence showed 100% similarity to the predicted sequence, confirming the accuracy of the results and ruling out sequencing errors. In conclusion, this study employed an optimized method to identify the prophage sequences of Liberibacter, achieving both high sensitivity and high accuracy.

The presence of prophages in Liberibacter species, particularly in CLas, provides insights into the diversity and evolution of Liberibacter. Previous studies have analyzed the genetic diversity of CLas across different regions based on the diversity of SC1, SC2, and P-JXGC-3 prophages [26]. In this study, we identified a more comprehensive set of prophages, offering a broader view of CLas diversity. Interestingly, we observed inconsistencies in the length and location of the same prophage across all CLas strains, such as NF1 (Table S3). This suggests that prophages located at the same position in different CLas strains may have originated from a single viral insertion event, indicating closer evolutionary relationships among these strains. By integrating analyses of prophage distribution and insertion site information, we can infer prophage integration patterns in the genome, facilitating evolutionary analysis of CLas strains.

Compared to inactive prophages, active prophages play a significant role in the co-evolution of bacteria and prophages, study of prophage physiological and biochemical mechanisms, and design of engineered phage applications [6,7]. In agriculture, engineered prophages can help reduce the population of plant pathogenic bacteria [42]. For example, the activation of active prophages can lyse plant pathogens, control disease outbreaks, and reduce the use of chemical pesticides, providing an environmentally friendly approach to disease management. Through genetic engineering techniques, virulence genes in prophages can be replaced with plant disease resistance genes or antimicrobial peptide genes, achieving the goal of disease control [43]. Engineered phages delivering CRISPR-AsCas12f1 targeting the *hrpB* gene of *Ralstonia solanacearum* can effectively control bacterial wilt disease in plants [44].

In this study, we found that most Type 4 phages are active and may potentially be activated for use as a control strategy against CLas. Additionally, Type 4 phages carry various virulence genes, including a shared core set of virulence genes, laying the groundwork for their engineered modification. The discovery of Type 4 prophages highlights the importance of comprehensively understanding the dynamics between prophages and their hosts, which is critical for elucidating bacterial ecology, the evolution of pathogenicity, and the development of innovative antimicrobial strategies.

## 5. Conclusions

This study highlights the diversity and potential roles of prophages in CLas genomes. We identified 191 prophages across 48 strains, including 13 novel types, and found that prophage integration impacts key biological processes such as genome structure, gene expression, and stress response. Virulence factors like carB and clpC were identified, suggesting a role in the global spread of CLas. Importantly, 19 out of 35 active Type 4 prophages offer promise for phage therapy against HLB. This research enhances our understanding of Liberibacter diversity and suggests new approaches for controlling HLB.

## Figures and Tables

**Table 1:** The selected 12 representative prophages.

NO.	Prophage	Species	Start	End	Length	Category	GC	Gene Number
1	Liberibacter phage NF1	CLas_LBR19TX2	85,461	127,782	42,322	Inactive	35.93%	39
2	Liberibacter phage NF2	CLas_A4	35,088	64,469	29,382	Inactive	35.94%	25
3	Liberibacter phage NF3	CLas_A4	169,378	196,298	26,921	Inactive	37.01%	24
4	Liberibacter phage NF4	CLas_JXGZ-1	217,913	248,206	30,294	Inactive	35.64%	30
5	Liberibacter phage NF5	CLso_ZC1	508,453	554,221	45,769	Inactive	35.65%	37
6	Liberibacter phage NF6	Lcr_BT-0	384,631	421,836	37,206	Inactive	33.77%	29
7	Liberibacter phage NF7	Lcr_BT-0	1,129,261	1,168,113	38,853	Inactive	33.48%	31
8	Liberibacter phage NF8	Lcr_BT-1	1,133,653	1,157,569	23,917	Inactive	33.59%	26
9	Liberibacter phage NF9	CLso_ZC1	326,297	355,216	28,920	Inactive	34.56%	22
10	Liberibacter phage NF10	CLas_SGCA16	1	24,286	24,286	Inactive	34.96%	15
11	Liberibacter phage NF11	CLas_YNJS7C	1,003,695	1,024,766	21,072	Inactive	34.49%	18
12	Liberibacter phage NF12	CLas_TX2351	14,137	54,885	40,749	Inactive	35.92%	37
13	Liberibacter phage NF13	CLas_HHCA	5560	20,683	15,124	Inactive	36.76%	15

**Table 2:** Virulence factors identified in this study.

Prophage Name	Virulence Factor	Gene ID	Description	Pident	E-Value	Bitscore
NF1	*carB*	VFG047708	carbamoyl phosphate synthase large subunit	53.6	0	1160
NF2	*clpC*	VFG000079	endopeptidase Clp ATP-binding chain C	46.0	1.24 × 10^−241^	695
NF3	*carB*	VFG047708	carbamoyl phosphate synthase large subunit	53.6	0	1160
NF4	*carB*	VFG047708	carbamoyl phosphate synthase large subunit	53.6	0	1160
NF5	*flhA*	VFG016784	flagellar biosynthesis protein	56.3	1.46 × 10^−^^257^	724
NF6	*algW*	VFG014984	AlgW protein	45.5	4.25 × 10^−^^72^	230
NF7	*rffG*	VFG013368	dTDP-glucose 46-dehydratase	54.9	1.39 × 10^−^^133^	382
NF8	*IlpA*	VFG045346	immunogenic lipoprotein A	42.4	2.19 × 10^−^^66^	204
NF10	*clpC*	VFG000079	endopeptidase Clp ATP-binding chain C	45.2	2.16 × 10^−^^237^	684
NF12	*carB*	VFG047708	carbamoyl phosphate synthase large subunit	56.4	0	1050
NF13	*clpC*	VFG000079	endopeptidase Clp ATP-binding chain C	45.0	8.64 × 10^−^^237^	682
Type1	*carB*	VFG047708	carbamoyl phosphate synthase large subunit	53.6	0	1160
Type2	*carB*	VFG047708	carbamoyl phosphate synthase large subunit	53.6	0	1160
Type3	*carB*	VFG047708	carbamoyl phosphate synthase large subunit	53.6	0	1160
Type4	*carB*	VFG047708	carbamoyl phosphate synthase large subunit	53.6	0	1160

**Table 3:** Type 4 prophages identified in this study.

NO.	Strain	Start	End	Length	Category	Score	Gene Number
1	CLaf_Ang37	25,421	60,648	35,228	Ambiguous	0.69	33
2	CLaf_PTSAPSY	25,432	60,671	35,240	Active	0.81	38
3	CLas_A4	1,126,504	1,157,896	31,393	Active	0.83	40
4	CLas_AHCA1	1,126,566	1,160,514	33,949	Active	0.86	42
5	CLas_AHCA17	2062	23,273	21,212	Active	0.96	29
6	CLas_CoFLP	1,126,499	1,157,891	31,393	Active	0.84	41
7	CLas_DUR1TX1	1660	14,578	12,919	Active	0.96	23
8	CLas_FL17	1,128,723	1,160,115	31,393	Active	0.86	42
9	CLas_GFR3TX3	4166	25,372	21,207	Active	0.96	30
10	CLas_gxpsy	1,114,937	1,148,879	33,943	Ambiguous	0.8	44
11	CLas_HHCA16	1146	13,703	12,558	Active	0.92	20
12	CLas_Ishi-1	1,124,179	1,155,572	31,394	Active	0.82	39
13	CLas_JRPAMB1	711,116	742,508	31,393	Active	0.83	41
14	CLas_JXGC	1,126,509	1,160,448	33,940	Ambiguous	0.8	44
15	CLas_JXGZ-1	33,780	67,719	33,940	Ambiguous	0.79	45
16	CLas_LBR19TX2	25,703	79,163	53,461	Inactive	0.38	59
17	CLas_LBR23TX5	45,412	76,804	31393	Active	0.83	40
18	CLas_Mex8	3426	24,493	21,068	Ambiguous	0.79	27
19	CLas_MFL16	812	18,998	18,187	Active	0.9	30
20	CLas_PA19	714	31,808	31,095	Ambiguous	0.65	41
21	CLas_PA20	714	31,808	31,095	Ambiguous	0.67	40
22	CLas_psy62	1,128,652	1,160,042	31,391	Active	0.85	42
23	CLas_ReuSP1	1,126,150	1,157,542	31,393	Active	0.83	40
24	CLas_SGCA16	28,111	59,503	31,393	Active	0.83	41
25	CLas_TaiYZ2	1,126,514	1,157,907	31,394	Active	0.84	41
26	CLas_TX2351	18	11,113	11,096	Active	0.89	19
27	CLas_YCPsy	688,253	722,192	33,940	Ambiguous	0.8	45
28	CLas_YNJS7C	1,122,402	1,153,794	31,393	Active	0.84	41
29	CLas_YNXP-1	63	26,166	26,104	Ambiguous	0.54	33
30	CLso_FIN111	66,818	111,284	44,467	Inactive	0.34	39
31	CLso_FIN114	36,023	77,833	41,811	Inactive	0.41	39
32	CLso_R1	2452	22,251	19,800	Ambiguous	0.72	24
33	CLso_ZC1	800,958	839,738	38,781	Inactive	0.33	37
34	Lcr_BT-0	885,577	922,825	37,249	Inactive	0.36	41
35	Lcr_BT-1	831,656	886,627	54,972	Inactive	0.24	66

## Data Availability

The 93 prophage genomes, CDS sequences, and amino acid sequences of the 13 novel phages identified in this study are accessible in the Science Data Bank (https://www.scidb.cn/en/s/jIJZfa) (accessed on 18 May 2025).

## Supplementary Materials

The following supporting information can be downloaded at: https://www.mdpi.com/article/10.3390/biology14050576/s1, Figure S1: PCR validation of the four prophages in Lcr str. BT-1. A: PCR amplified fragments of hallmark gene on agarose gel. B: PCR validation of hallmark genes in bacterial cultures. The fragment lengths of NF2, NF6, NF7, and NF8 were 861 bp, 531 bp, 2619 bp, and 1473 bp, respectively. The bands indicated by arrows in A and B were target bands; Table S1: The Liberibacter genome used in this study; Table S2: The diversity of prophage in Liberibacter species; Table S3: Information of all identified prophages.
