# Supplementary material for: Unveiling Prophage Diversity and Host Interactions in Liberibacter: Genomic Insights for Phage Therapy Against Citrus Huanglongbing
Source: Biology (Basel). 2025 May 20;14(5):576. doi: 10.3390/biology14050576 (PMC12109144; doi:10.3390/biology14050576)
Supplement: Supplementary file 1 [file biology-14-00576-s001.zip › SI-figure1.pdf]

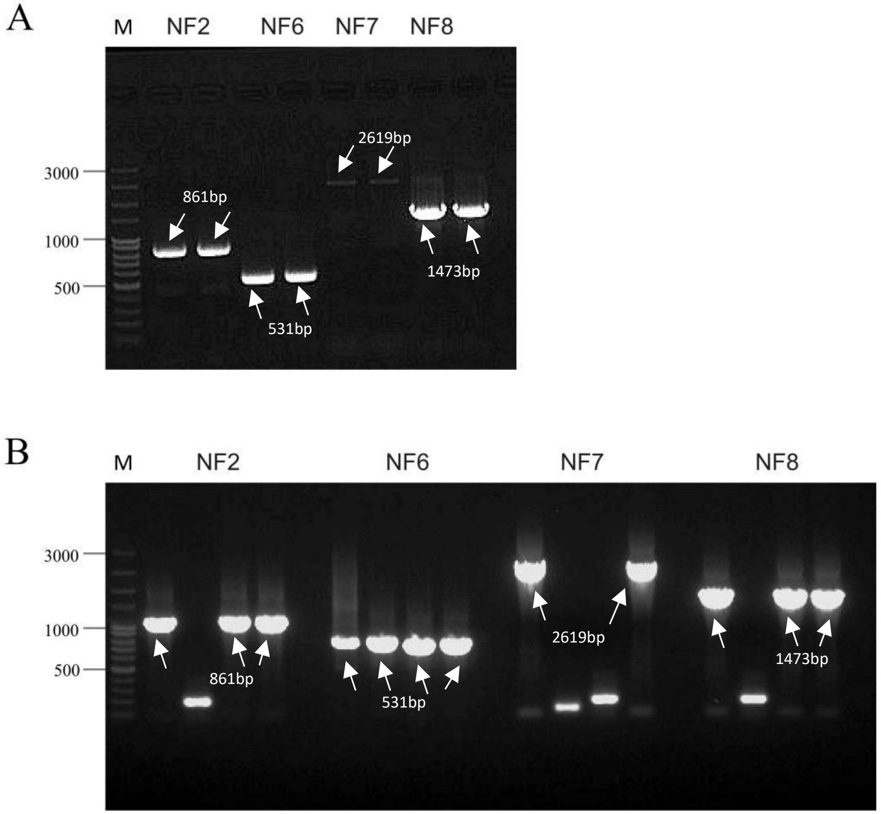

Figure S1 PCR validation of the four prophages in Lcr str. BT-1. A: PCR amplified fragments of hallmark gene on agarose gel. B: PCR validation of hallmark genes in bacterial cultures.The fragment lengths of NF2, NF6, NF7 and NF8 were 861bp, 531bp, 2619bp and 1473bp, respectively. The bands indicated by arrows in A and B were target bands.
