# Supplementary material for: Unveiling Prophage Diversity and Host Interactions in Liberibacter: Genomic Insights for Phage Therapy Against Citrus Huanglongbing
Source: Biology (Basel). 2025 May 20;14(5):576. doi: 10.3390/biology14050576 (PMC12109144; doi:10.3390/biology14050576)
Supplement: Supplementary file 1 [file biology-14-00576-s001.zip › SI-table.pdf]

TABLE S1 The *Liberibacter* genome used in this study

Number	Species	Strain	Accession number	Level	Size （Mb)	Gene Count	Country
1	CLas	9PA	GCA_013778575.1	Contig	1.23	1224	Brazil: Sao Paulo
2	CLas	CHUC	GCA_009756785.1	Contig	1.21	1095	China
3	CLas	YCPsy	GCA_001296945.1	Contig	1.23	1113	China: Guangdong
4	CLas	gxpsy	GCA_000346595.1	Chromosome	1.27	1191	China: guangxi
5	CLas	A4	GCA_000590865.3	Chromosome	1.23	1123	China: guanzhou
6	CLas	JXGC	GCA_002216815.1	Chromosome	1.23	1118	China: Jiangxi
7	CLas	JXGZ-1	GCF_009764765.1	Contig	1.22	1095	China: JiangXi
8	CLas	YNXP-1	GCF_009764755.1	Contig	1.21	1094	China: YunNan
9	CLas	YNJS7C	GCA_003615235.1	Contig	1.26	1158	China:Yunnan
10	CLas	CoFLP	GCA_014107775.1	Chromosome	1.23	1104	Colombia: Municipio Dibulla
11	CLas	ReuSP1	GCA_022220845.1	Chromosome	1.23	1099	France: La Reunion
12	CLas	Ishi-1	GCA_000829355.1	Chromosome	1.20	1127	Japan: Okinawa, Ishigaki
13	CLas	Mex8	GCA_009756755.1	Contig	1.24	1131	Mexico: Mexicali
14	CLas	PA19	GCF_013309695.2	Contig	1.22	1106	Pakistan
15	CLas	PA20	GCF_016758155.2	Contig	1.23	1109	Pakistan
16	CLas	TaiYZ2	GCA_014217975.1	Chromosome	1.23	1123	Thailand:Songkhla
17	CLas	psy62	GCA_000023765.2	Complete	1.23	1162	USA
18	CLas	AHCA1	GCA_003143875.1	Chromosome	1.23	1112	USA: Anaheim, California
19	CLas	A-SBCA19	GCA_014892655.1	Contig	1.19	1115	USA: California
20	CLas	AHCA17	GCF_009859045.1	Scaffold	1.21	1097	USA: California
21	CLas	SGCA5	GCA_001430705.1	Contig	1.20	1100	USA: California, San Gabriel
22	CLas	CRCFL16	GCA_009756805.1	Contig	1.21	1091	USA: Florida

23	CLas	MFL16	GCA_009756815.1	Contig	1.20	1081	USA: Florida
24	CLas	JRPAMB1	GCA_013462975.1	Chromosome	1.24	1128	USA: Florida
25	CLas	HHCA16	GCA_009756845.1	Contig	1.21	1101	USA: Hacienda Heights
26	CLas	HHCA	GCF_000724755.1	Contig	1.15	1199	USA: Los Angeles
27	CLas	SGCA16	GCA_009756855.1	Contig	1.21	1098	USA: San Gabriel
28	CLas	TX2351	GCA_001969535.1	Contig	1.25	1184	USA: Texas
29	CLas	DUR2TX1	GCA_009756725.1	Contig	1.21	1117	USA: Texas
30	CLas	GFR3TX3	GCA_009756735.1	Contig	1.21	1092	USA: Texas
31	CLas	DUR1TX1	GCA_009756745.1	Contig	1.21	1091	USA: Texas
32	CLas	LBR19TX2	GCA_009756885.1	Contig	1.20	1085	USA: Texas
33	CLas	LBR23TX5	GCA_009756915.1	Contig	1.20	1089	USA: Texas
34	CLas	FL17	GCA_000820625.1	Contig	1.23	1103	USA:Florida
35	CLaf	Ang37	GCF_017869345.1	Chromosome	1.19	1138	Angola
36	CLaf	PTSAPSY	GCF_001021085.1	Complete	1.19	1101	Pretoria
37	CLam	PW_SP	GCF_000350385.1	Contig	1.18	1019	Brazil
38	CLam	Sao Paulo	GCF_000496595.1	Complete	1.20	1046	Brazil
39	CLso	CLso-ZC1	GCF_000183665.1	Complete	1.26	1164	USA: Dalhart, Texas
40	CLso	LsoNZ1	GCF_000968085.1	Contig	1.31	1210	New Zealand
41	CLso	FIN114	GCF_001983675.1	Contig	1.25	1154	Finland: Forssa
42	CLso	HenneA	GCF_000968075.1	Contig	1.21	1144	USA: Weslaco, TX
43	CLso	RSTM	GCF_001414235.1	Contig	1.29	1209	USA: California
44	CLso	FIN111	GCF_001983655.1	Contig	1.20	1103	Finland: Forssa
45	CLso	ISR100	GCF_002918245.2	Contig	1.30	1228	Israel
46	CLso	R1	GCF_000756225.1	Contig	1.20	1142	USA:California
47	Lcr	BT-0	GCF_001543305.1	Complete	1.52	1389	/
48	Lcr	BT-1	GCF_000325745.1	Complete	1.50	1388	/

TABLE S2 The diversity of prophage in *Liberibacter* species

Prophage name	NF1	NF2	NF3	NF4	NF5	NF6	NF7	NF8	NF9	NF10	NF11	NF12	NF13	Type 1	Type 2	Type 3	Type 4	
Closest virus	Aeromonas phage 31.2	Agrobacterium phage Atu_ph07	Pelagibacter phage HTVC008M	Streptococcus phage phi1207.3	Aeromonas phage 44RR2.8t.2	Acinetobacter phage AB1	Sphingomonas phage PAU	Synechococcus phage S-WAM1	Aureococcus anophagefferens virus	Bacillus phage proCM3	Nodularia_phage_vB_NpeS-2AV2	Prochlorococcus phage P-SSM2	Streptococcus phage Dp-1	Liberibacter phage SC1	Liberibacter phage SC2	Liberibacter phage P-JXGC-3	Xylella phage Xfas53	Total
CLaf_Ang37	+													+	+		+	4
CLaf_PTSAPSY	+													+	+		+	4
CLam_PW_SP														+	+			2
CLam_Sao Paulo		+													+			2
CLas_9PA	+															+		2
CLas_A4	+	+	+	+											+		+	6
CLas_AHCA1	+	+	+	+										+	+		+	7
CLas_AHCA17														+			+	2
CLas_A-SBCA19	+																	1
CLas_CHUC	+													+	+			3
CLas_CoFLP	+	+	+	+										+			+	6
CLas_CRCFL16														+				1
CLas_DUR1TX1														+			+	2
CLas_DUR2TX1														+	+			2
CLas_FL17	+	+	+	+										+	+	+	+	8
CLas_GFR3TX3	+																+	2

CLas_gxpsy	+	+	+	+										+	+	+	7
CLas_HHCA		+										+		+			3
CLas_HHCA16			+	+												+	3
CLas_Ishi-1	+	+	+	+											+	+	6
CLas_JRPAMB1	+	+	+										+	+		+	6
CLas_JXGC	+	+	+	+											+	+	6
CLas_JXGZ-1				+									+	+	+	+	5
CLas_LBR19TX2	+												+			+	3
CLas_LBR23TX5				+									+			+	3
CLas_Mex8														+		+	2
CLas_MFL16																+	1
CLas_PA19			+	+										+		+	4
CLas_PA20		+	+	+										+		+	5
CLas_psy62	+	+	+	+									+			+	6
CLas_ReuSP1	+	+	+	+									+			+	6
CLas_SGCA5				+									+				2
CLas_SGCA16	+								+							+	3
CLas_TaiYZ2	+	+	+	+										+		+	6
CLas_TX2351	+		+								+		+			+	5
CLas_YCPsy	+	+	+										+			+	5
CLas_YNJS7C	+	+	+	+						+				+	+	+	8
CLas_YNXP-1			+	+										+		+	4
CLso_FIN111													+	+		+	3
CLso_FIN114					+								+	+		+	4
CLso_HenneA													+	+			2
CLso_ISR100													+	+			2

CLso_LsoNZ1		+												+	+	+		4
CLso_R1					+									+			+	3
CLso_RSTM					+									+	+			3
CLso_ZC1		+			+				+					+	+	+	+	7
Lcr_BT-0		+				+	+	+									+	5
Lcr_BT-1		+				+	+	+									+	5
Total	22	20	18	18	4	2	2	2	1	1	1	1	1	28	26	9	35	191

TABLE S3 Information of all identified prophages

Prophage	Class	Species	Start	End	Length	Category	Score	GC	Gene number
NF1	Caudoviricetes	CLaf_Ang37	254,841	320,350	65,510	Inactive	0.05	35.14%	57
		CLaf_PTSAPSY	254,895	320,415	65,521	Inactive	0.04	35.13%	54
		CLas_9PA	6,778	45,578	38,801	Inactive	0.02	36.66%	31
		CLas_A4	230,123	248,969	18,847	Inactive	0.01	36.36%	17
		CLas_AHCA1	230,129	248,975	18,847	Inactive	0.01	36.36%	17
		CLas_A-SBCA19	98	27,864	27,767	Inactive	0.02	35.95%	25
		CLas_CHUC	321	33,881	33,561	Inactive	0.02	36.61%	28
		CLas_CoFLP	229,952	248,798	18,847	Inactive	0.01	36.36%	17
		CLas_FL17	236,386	255,232	18,847	Inactive	0.01	36.37%	17
		CLas_GFR3TX3	54,727	73,573	18,847	Inactive	0.01	36.37%	17
		CLas_gxpsy	222,884	241,730	18,847	Inactive	0.02	36.37%	17
		CLas_Ishi-1	228,538	247,384	18,847	Inactive	0.01	36.38%	17
		CLas_JRPAMB1	380,772	408,552	27,781	Inactive	0.02	35.95%	25
		CLas_JXGC	230,104	248,950	18,847	Inactive	0.01	36.37%	17
		CLas_LBR19TX2	85,461	127,782	42,322	Inactive	0.03	35.93%	39
		CLas_psy62	236,343	255,189	18,847	Inactive	0.01	36.37%	17
		CLas_ReuSP1	229,951	248,797	18,847	Inactive	0.01	36.35%	17
		CLas_SGCA16	90,862	128,649	37,788	Inactive	0.03	36.10%	32
		CLas_TaiYZ2	230,123	248,969	18,847	Inactive	0.01	36.36%	17
		CLas_TX2351	101,671	120,517	18,847	Inactive	0.02	36.38%	18
		CLas_YCPsy	226,791	254,557	27,767	Inactive	0.02	35.96%	25
		CLas_YNJS7C	230,123	248,969	18,847	Inactive	0.01	36.36%	17
NF2	Caudoviricetes	CLam_Sao Paulo	1,108,770	1,138,358	29,589	Inactive	0.11	28.95%	27

		CLas_A4	35,088	64,469	29,382	Inactive	0.05	35.94%	25
		CLas_AHCA1	35,093	64,474	29,382	Inactive	0.04	35.94%	24
		CLas_CoFLP	34,917	64,298	29,382	Inactive	0.04	35.94%	24
		CLas_FL17	41,346	70,727	29,382	Inactive	0.04	35.94%	24
		CLas_gxpsy	27,843	57,224	29,382	Inactive	0.05	35.95%	25
		CLas_HHCA	4	10,343	10,340	Inactive	0.03	37.23%	7
		CLas_Ishi-1	33,536	62,917	29,382	Inactive	0.04	35.94%	24
		CLas_JRPAMB1	574,185	603,566	29,382	Inactive	0.05	35.94%	25
		CLas_JXGC	35,069	64,450	29,382	Inactive	0.05	35.95%	25
		CLas_PA20	33,485	62,872	29,388	Inactive	0.04	35.94%	24
		CLas_psy6	41,336	70,717	29,382	Inactive	0.04	35.94%	24
		CLas_ReuSP1	34,917	64,298	29,382	Inactive	0.05	35.94%	25
		CLas_TaiYZ2	35,088	64,469	29,382	Inactive	0.05	35.95%	25
		CLas_YCPsy	31,749	61,130	29,382	Inactive	0.05	35.95%	25
		CLas_YNJS7C	35,089	64,470	29,382	Inactive	0.05	35.95%	25
		CLso_LsoNZ1	201,837	220,560	18,724	Inactive	0.17	33.34%	23
		CLso_ZC1	966,146	1,002,749	36,604	Inactive	0.02	34.35%	33
		Lcr_BT-0	1,006,865	1,035,765	28,901	Ambiguous	0.59	37.16%	38
		Lcr_BT-1	302,709	326,032	23,324	Inactive	0	34.88%	16
		CLas_A4	169,378	196,298	26,921	Inactive	0.16	37.01%	24
		CLas_AHCA1	169,383	196,303	26,921	Inactive	0.15	37.01%	24
		CLas_CoFLP	169,207	196,127	26,921	Inactive	0.15	37.01%	24
NF3	Caudoviricetes	CLas_FL17	175,640	202,560	26,921	Inactive	0.15	37%	24
		CLas_gxpsy	162,139	189,059	26,921	Inactive	0.15	37%	24
		CLas_HHCA16	3	22,389	22,387	Inactive	0.15	36.83%	21
		CLas_Ishi-1	167,799	194,714	26,916	Inactive	0.16	37%	23

		CLas_JRPAMB1	442,384	469,304	26,921	Inactive	0.15	37%	24
		CLas_JXGC	169,359	196,279	26,921	Inactive	0.15	37%	24
		CLas_PA19	167,774	194,688	26,915	Inactive	0.15	36.99%	24
		CLas_PA20	167,782	194,697	26,916	Inactive	0.15	36.99%	24
		CLas_psy62	175,600	202,518	26,919	Inactive	0.16	37%	24
		CLas_ReuSP1	169,206	196,126	26,921	Inactive	0.15	37%	24
		CLas_TaiYZ2	169,378	196,298	26,921	Inactive	0.15	37%	24
		CLas_TX2351	40,919	67,840	26,922	Inactive	0.16	37.01%	24
		CLas_YCPsy	166,039	192,959	26,921	Inactive	0.15	37%	24
		CLas_YNJS7C	169,378	196,298	26,921	Inactive	0.16	37%	24
		CLas_YNXP-1	58,446	85,366	26,921	Inactive	0.16	37%	24
		CLas_A4	748,323	766,698	18,376	Inactive	0.07	34.36%	21
		CLas_AHCA1	748,368	766,743	18,376	Inactive	0.07	34.36%	21
		CLas_CoFLP	748,262	766,637	18,376	Inactive	0.07	34.36%	21
		CLas_FL17	754,596	772,970	18,375	Inactive	0.07	34.46%	21
		CLas_gxpsy	739,119	757,494	18,376	Inactive	0.07	34.36%	21
		CLas_HHCA16	905	24,196	23,292	Inactive	0.07	35.85%	24
		CLas_Ishi-1	746,638	765,013	18,376	Inactive	0.07	34.36%	21
NF4	Caudoviricetes	CLas_JXGC	748,319	766,694	18,376	Inactive	0.07	34.36%	21
		CLas_JXGZ-1	217,913	248,206	30,294	Inactive	0.07	35.64%	30
		CLas_LBR23TX5	139	34,373	34,235	Inactive	0.08	35.24%	34
		CLas_PA19	337,941	356,316	18,376	Inactive	0.06	34.37%	20
		CLas_PA20	338,689	357,064	18,376	Inactive	0.07	34.37%	21
		CLas_psy62	754,522	772,896	18,375	Inactive	0.07	34.36%	21
		CLas_ReuSP1	747,972	766,347	18,376	Inactive	0.07	34.36%	21
		CLas_SGCA5	81	34,314	34,234	Inactive	0.09	35.25%	35

		CLas_TaiYZ2	748,335	766,710	18,376	Inactive	0.07	34.35%	21
		CLas_YNJS7C	748,296	766,671	18,376	Inactive	0.07	34.36%	21
		CLas_YNXP-1	75,276	105,569	30,294	Inactive	0.06	35.64%	30
		CLso_FIN114	497,725	551,695	53,971	Inactive	0.05	35.26%	47
NF5	Caudoviricetes	CLso_R1	75	68,816	68,742	Inactive	0.05	35.33%	57
		CLso_RSTM	51,655	124,390	72,736	Inactive	0.05	35.11%	61
		CLso_ZC1	508,453	554,221	45,769	Inactive	0.03	35.65%	37
NF6	Caudoviricetes	Lcr_BT-0	384,631	421,836	37,206	Inactive	0.02	33.77%	29
		Lcr_BT-1	342,990	380,195	37,206	Inactive	0.02	33.77%	29
NF7	Caudoviricetes	Lcr_BT-0	1,129,261	1,168,113	38,853	Inactive	0.01	33.48%	31
		Lcr_BT-1	1,075,574	1,114,422	38,849	Inactive	0.01	33.48%	32
NF8	Caudoviricetes	Lcr_BT-0	1,187,856	1,215,382	27,527	Inactive	0.03	33.76%	24
		Lcr_BT-1	1,133,653	1,157,569	23,917	Inactive	0.06	33.59%	26
NF9	Megaviricetes	CLso_ZC1	326,297	355,216	28,920	Inactive	0.06	34.56%	22
NF10	Caudoviricetes	CLas_SGCA16	1	24,286	24,286	Inactive	0.01	34.96%	15
NF11	/	CLas_YNJS7C	1,003,695	1,024,766	21,072	Inactive	0.17	34.49%	18
NF12	Caudoviricetes	CLas_TX2351	14,137	54,885	40,749	Inactive	0.09	35.92%	37
NF13	Caudoviricetes	CLas_HHCA	5,560	20,683	15,124	Inactive	0.04	36.76%	15
		CLaf_Ang37	449,409	489,710	40,302	Ambiguous	0.74	39.64%	44
		CLaf_PTSAPSY	449,492	489,789	40,298	Ambiguous	0.76	39.63%	48
		CLam_PW_SP	9,658	25,501	15,844	Inactive	0.44	31.52%	17
Type 1	Caudoviricetes	CLas_AHCA1	1,198,204	1,227,869	29,666	Active	0.95	38.15%	14
		CLas_AHCA17	68,287	98,367	30,081	Inactive	0.03	36.45%	25
		CLas_CHUC	8,268	37,345	29,078	Inactive	0.48	42.39%	24
		CLas_CoFLP	1,179,847	1,200,840	20,994	Inactive	0.44	39.19%	23
		CLas_CRCFL16	252	15,572	15,321	Inactive	0.39	44.42%	11

		CLas_DUR1TX1	620	11,131	10,512	Ambiguous	0.8	43.41%	8
		CLas_DUR2TX1	10	19,794	19,785	Inactive	0.09	36.03%	21
		CLas_FL17	1,162	13,267	12,106	Ambiguous	0.74	39.28%	12
		CLas_JRPAMB1	634,371	661,468	27,098	Ambiguous	0.74	39.35%	33
		CLas_JXGZ-1	3,469	36,158	32,690	Inactive	0.03	36.82%	26
		CLas_LBR19TX2	860	17,655	16,796	Inactive	0.26	44.24%	10
		CLas_LBR23TX5	648	11,159	10,512	Ambiguous	0.8	43.44%	8
		CLas_psy62	1,210,272	1,226,454	16,183	Ambiguous	0.76	40.57%	17
		CLas_ReuSP1	1,179,498	1,199,411	19,914	Ambiguous	0.55	39.09%	20
		CLas_SGCA5	2,342	17,663	15,322	Inactive	0.38	44.42%	11
		CLas_TX2351	527	11,644	11,118	Inactive	0.16	36.51%	11
		CLas_YCPsy	1,990	22,016	20,027	Ambiguous	0.61	43.48%	16
		CLso_FIN111	444	11,894	11,451	Ambiguous	0.79	33.32%	9
		CLso_FIN114	431,820	448,906	17,087	Ambiguous	0.53	34.54%	19
		CLso_HenneA	130,955	165,348	34,394	Inactive	0.29	35.75%	42
		CLso_ISR100	1,255	21,546	20,292	Ambiguous	0.52	34.06%	18
		CLso_LsoNZ1	311,576	345,969	34,394	Inactive	0.24	35.76%	43
		CLso_R1	34	19,328	19,295	Ambiguous	0.63	41.75%	15
		CLso_RSTM	266,207	298,458	32,252	Inactive	0.18	36.27%	38
		CLso_ZC1	17,348	38,272	20,925	Inactive	0.29	33.77%	26
		CLaf_Ang37	1,092,247	1,119,432	27,186	Ambiguous	0.52	32.39%	35
		CLaf_PTSAPSY	1,087,704	1,115,639	27,936	Inactive	0.45	32.73%	29
		CLam_PW_SP	983	36,439	35,457	Inactive	0.22	29.82%	33
Type 2	Caudoviricetes	CLam_Sao Paulo	749,389	793,654	44,266	Inactive	0.48	36.87%	45
		CLas_A4	1,182,998	1,214,074	31,077	Inactive	0.48	38.96%	29
		CLas_AHCA1	1,185,617	1,216,971	31,355	Active	0.84	39.38%	17

		CLas_CHUC	19	17,138	17,120	Ambiguous	0.68	40.10%	21
		CLas_DUR2TX1	362	19,509	19,148	Ambiguous	0.51	39.95%	19
		CLas_FL17	1,181,795	1,195,691	13,897	Ambiguous	0.56	38.48%	12
		CLas_gxpsy	1,188,334	1,225,253	36,920	Ambiguous	0.64	39.28%	39
		CLas_HHCA	523	13,070	12,548	Active	0.84	40.96%	17
		CLas_JRPAMB1	654,471	686,014	31,544	Ambiguous	0.6	39.21%	30
		CLas_JXGZ-1	544	18,394	17,851	Inactive	0.01	36.29%	11
		CLas_Mex8	1,674	17,455	15,782	Ambiguous	0.77	39.61%	14
		CLas_PA19	359	29,973	29,615	Ambiguous	0.68	39.05%	32
		CLas_PA20	15	36,883	36,869	Ambiguous	0.66	39.06%	40
		CLas_TaiYZ2	1,183,009	1,214,471	31,463	Inactive	0.48	39.05%	29
		CLas_YNJS7C	6,659	39,567	32,909	Ambiguous	0.71	41.77%	34
		CLas_YNXP-1	47	17,080	17,034	Inactive	0.45	39.08%	13
		CLso_FIN111	19,722	34,776	15,055	Ambiguous	0.55	33.05%	17
		CLso_FIN114	158,118	206,608	48,491	Ambiguous	0.68	39.85%	60
		CLso_HenneA	42,797	72,920	30,124	Inactive	0.31	35.68%	34
		CLso_ISR100	18	10,177	10,160	Inactive	0.11	34.42%	8
		CLso_LsoNZ1	9,992	63,743	53,752	Ambiguous	0.63	36.17%	62
		CLso_RSTM	26	41,059	41,034	Inactive	0.36	33.02%	41
		CLso_ZC1	175,469	216,842	41,374	Ambiguous	0.71	40.42%	55
		CLas_9PA	24,635	41,418	16,784	Ambiguous	0.8	41.19%	18
		CLas_FL17	358	12,821	12,464	Ambiguous	0.68	39.54%	19
		CLas_gxpsy	1,231,493	1,267,088	35,596	Ambiguous	0.69	41.08%	37
Type 3	Caudoviricetes	CLas_Ishi-1	1,176,645	1,190,536	13,892	Ambiguous	0.63	38.13%	12
		CLas_JXGC	1,202,176	1,225,060	22,885	Ambiguous	0.55	40.61%	22
		CLas_JXGZ-1	1,408	12,290	10,883	Inactive	0.05	41.18%	8

		CLas_YNJS7C	3,803	31,518	27,716	Inactive	0.47	40.45%	29
		CLso_LsoNZ1	107,970	143,914	35,945	Inactive	0.09	34.23%	34
		CLso_ZC1	1,232,493	1,257,872	25,380	Active	0.87	38.45%	39
		CLaf_Ang37	25,421	60,648	35,228	Ambiguous	0.69	33.48%	33
		CLaf_PTSAPSY	25,432	60,671	35,240	Active	0.81	33.49%	38
		CLas_A4	1,126,504	1,157,896	31,393	Active	0.83	35%	40
		CLas_AHCA1	1,126,566	1,160,514	33,949	Active	0.86	35%	42
		CLas_AHCA17	2,062	23,273	21,212	Active	0.96	34.98%	29
		CLas_CoFLP	1,126,499	1,157,891	31,393	Active	0.84	35%	41
		CLas_DUR1TX1	1,660	14,578	12,919	Active	0.96	35.50%	23
		CLas_FL17	1,128,723	1,160,115	31,393	Active	0.86	35%	42
		CLas_GFR3TX3	4,166	25,372	21,207	Active	0.96	34.96%	30
		CLas_gxpsy	1,114,937	1,148,879	33,943	Ambiguous	0.8	35.02%	44
		CLas_HHCA16	1,146	13,703	12,558	Active	0.92	34.80%	20
Type 4	Caudoviricetes	CLas_Ishi-1	1,124,179	1,155,572	31,394	Active	0.82	34.99%	39
		CLas_JRPAMB1	711,116	742,508	31,393	Active	0.83	34.98%	41
		CLas_JXGC	1,126,509	1,160,448	33,940	Ambiguous	0.8	35.01%	44
		CLas_JXGZ-1	33,780	67,719	33,940	Ambiguous	0.79	35.01%	45
		CLas_LBR19TX2	25,703	79,163	53,461	Inactive	0.38	35.53%	59
		CLas_LBR23TX5	45,412	76,804	31,393	Active	0.83	34.98%	40
		CLas_Mex8	3,426	24,493	21,068	Ambiguous	0.79	34.70%	27
		CLas_MFL16	812	18,998	18,187	Active	0.9	35.95	30
		CLas_PA19	714	31,808	31,095	Ambiguous	0.65	35.42%	41
		CLas_PA20	714	31,808	31,095	Ambiguous	0.67	35.43%	40
		CLas_psy62	1,128,652	1,160,042	31,391	Active	0.85	34.98%	42
		CLas_ReuSP1	1,126,150	1,157,542	31,393	Active	0.83	35%	40

CLas_SGCA16	28,111	59,503	31,393	Active	0.83	35.01%	41
CLas_TaiYZ2	1,126,514	1,157,907	31,394	Active	0.84	35%	41
CLas_TX2351	18	11,113	11,096	Active	0.89	34.42%	19
CLas_YCPsy	688,253	722,192	33,940	Ambiguous	0.8	35.01%	45
CLas_YNJS7C	1,122,402	1,153,794	31,393	Active	0.84	34.99%	41
CLas_YNXP-1	63	26,166	26,104	Ambiguous	0.54	36.09%	33
CLso_FIN111	66,818	111,284	44,467	Inactive	0.34	33.24%	39
CLso_FIN114	36,023	77,833	41,811	Inactive	0.41	33.18%	39
CLso_R1	2,452	22,251	19,800	Ambiguous	0.72	34.51%	24
CLso_ZC1	800,958	839,738	38,781	Inactive	0.33	33.51%	37
Lcr_BT-0	885,577	922,825	37,249	Inactive	0.36	41.78%	41
Lcr_BT-1	831,656	886,627	54,972	Inactive	0.24	42.26%	66
